# A Novel Technique Using Ryle's Tube for Colonic Decompression in Acute Colonic Pseudo-Obstruction

**DOI:** 10.7759/cureus.50020

**Published:** 2023-12-06

**Authors:** Khalid Al Shamousi, Ayat Idris, Masoud Salim Kashoob, Said A Al-Busafi

**Affiliations:** 1 Gastroenterology Unit, Department of Medicine, College of Medicine and Health Sciences, Sultan Qaboos University, Muscat, OMN; 2 Infectious Disease Unit, Department of Medicine, Sultan Qaboos University Hospital, Muscat, OMN; 3 Internal Medicine, Oman Medical Specialty Board, Muscat, OMN

**Keywords:** ryle's tube, conservative management, colonic decompression, acute colonic pseudo-obstruction, ogilvie's syndrome

## Abstract

Acute colonic pseudo-obstruction, also known as Ogilvie's syndrome, involves colon dilation without mechanical obstruction. It is conventionally treated with conservative measures such as fasting, nasogastric and rectal tube placement, correction of fluids and electrolytes, and, if necessary, use of neostigmine and colonic decompression through colonoscopy. Surgical intervention may be considered in severe cases. In this report, we present a case of acute colonic pseudo-obstruction where initial conservative management failed. The patient was successfully treated using a novel rectal tube insertion technique.

## Introduction

Sir Heneage Ogilvie first documented Ogilvie's syndrome, or acute colonic pseudo-obstruction (ACPO), in 1948. In his initial case report, he observed colonic obstruction in patients without diagnosed colonic abnormalities, later revealing carcinoma unrelated to the colon during exploratory laparotomy [1]. ACPO manifests as pathological colon dilation without mechanical obstruction, primarily affecting patients with severe comorbidities [2,3]. Diagnosis relies on clinical and radiological criteria, and treatment options range from conservative approaches to interventions such as acetylcholinesterase inhibitors (e.g., neostigmine), decompressive procedures (including colonoscopy), and, in extreme cases, surgery [2]. While decompressive procedures typically involve nasogastric and rectal tubes, the use of Ryle's tube for decompression has not been previously reported. This case details the successful management of ACPO through a novel rectal tube insertion technique involving the passage of a Ryle's tube through a guidewire under fluoroscopy.

## Case presentation

A 49-year-old male with a history of diabetes mellitus type 2 and stage IV advanced metastatic colonic adenocarcinoma refractory to multiple lines of chemotherapy on palliative chemotherapy presented to the hospital with a three-week history of progressive constipation, abdominal distention, nausea, and self-induced vomiting to relieve the symptoms. Physical examination showed significant abdominal distention and diminished bowel sounds with no tenderness, guarding rigidity, or palpable masses. The initial laboratory tests showed hemoglobin levels of 9.9 glL, WBC of 10.4X 10^9/L with neutrophil 7.7X 10^9/L, platelet count of 477 X 10^9/L with mildly raised CRP of 67 mg/l, normal liver function test, and normal renal function and electrolytes test (Table 1).

**Table 1:** The laboratory data of the patient.

Blood test	Result	Normal Range
Hemoglobin	9.9 glL	12-15.8 g/dl
White blood cells	10.4 X 10^9/L	3.5-9.1 X 10^9/L
Neutrophil count	7.7 X 10^9/L	1.45-7.5 X 10^9/L
Platelet count	477 X 10^9/L	150- 450 X 10^9/L
Alanine aminotransferase (ALT)	19 U/L	0-35 U/L
Aspartate aminotransferase (AST)	27 U/L	0-40 U/L
Alkaline phosphatase	91 U/L	33-96 U/L
Albumin	26 g/L	35-55 g/L
Total bilirubin	5 umol/L	8-18 umol/L
C-reactive protein (CRP)	67 mg/l	8-10 mg/L
Anion gap	5 mmol/L	5-17 mmol/L
Bicarbonate	35 mmol/L	22-33 mmol/L
Sodium	140 mmol/L	135-145 mmol/L
Potassium	4.3 mmol/L	3.5-5.5 mmol/L
Chloride	100 mmol/L	95-105 mmol/L
Urea	7.6 mmol/L	2.1-8.5 mmol/L
Creatinine	30 umol/L	60-114 umol/L

**Figure 1:**
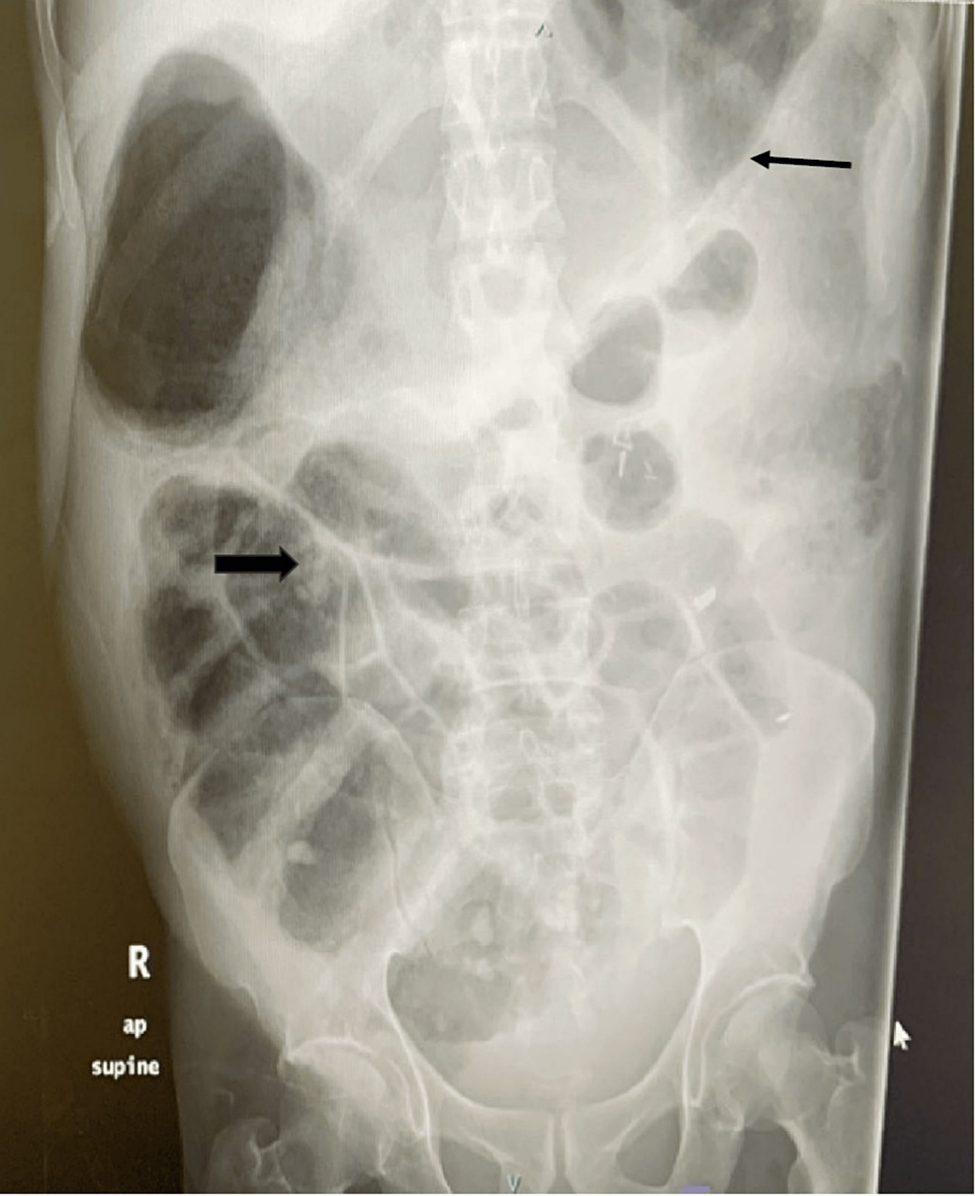
A plain abdomen (AP supine view) X-ray. The image shows the colon is loaded with fecal matter (block arrow) and grossly dilated, involving the ascending and transverse colon, reaching up to 9 cm in transverse (line arrow).

An abdomen X-ray displayed markedly dilated large bowel loops reaching up to 9 cm in the transverse colon with a fecal loading in the ascending colon (Figure 1).

Abdominal computerized tomography (CT) showed mild wall thickening at the distal anastomosis site with interval development of large bowel dilatation proximal to that level, particularly in the transverse and ascending colon levels. The largest diameter, measuring 9 cm, was noted at the level of the ascending colon containing diffuse fecal material, which could be due to partial obstruction at this level. 

**Figure 2:**
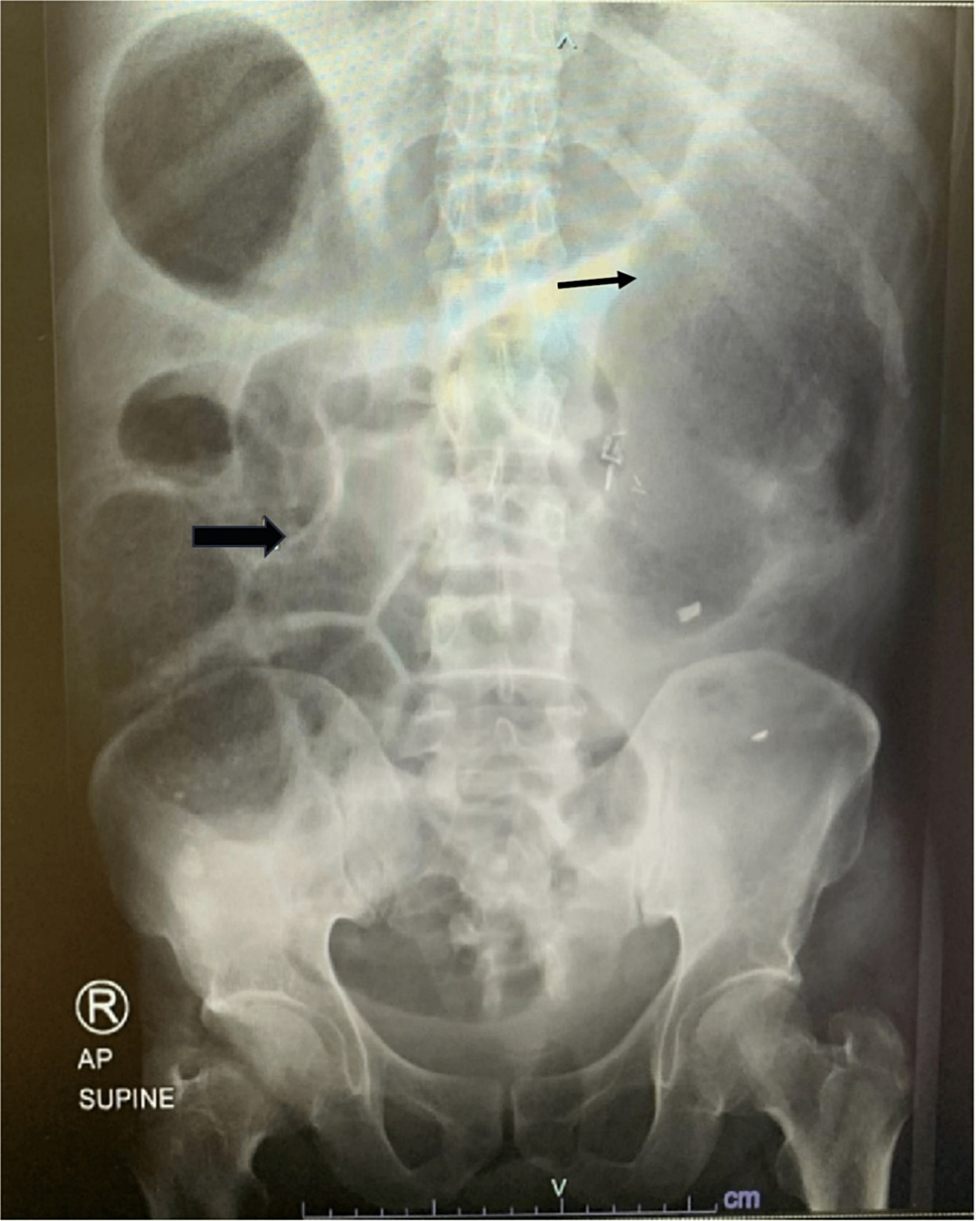
A follow-up plain abdominal (AP supine view) X-ray The image demonstrates a progression in distension of the large bowel loop with fecal matter (block arrow) with a probable transition point at the descending colon. The dilated descending colon measures approximately 13.5 cm in the maximum transverse diameter (line arrow).

The colorectal surgery team opted for conservative management, including nil per oral (NPO), nasogastric tube (NGT) insertion with low suction, intravenous hydration using normal saline, and laxative administration via NGT and enema. NuLYTELY (polyethylene glycol [PEG] 3350, sodium chloride, sodium bicarbonate, and potassium chloride for oral solution) was given orally (100 ml every 30 minutes for a total of 3 liters) and as an enema (250 ml was given at once). However, two days later, with no improvement observed, the patient underwent a colonoscopy for colonic decompression and potential stenting. The colonoscopy revealed no anastomotic stenosis, with the scope advancing up to 50 cm and a rectal tube placed up to the splenic flexure. Although there was initial improvement with reduced nausea and abdominal distension, the patient complained again of the same symptoms. An abdominal X-ray showed a large bowel loop dilatation progression, reaching a maximum diameter at a descending colon size of 13.5 cm (Figure 2).

**Figure 3:**
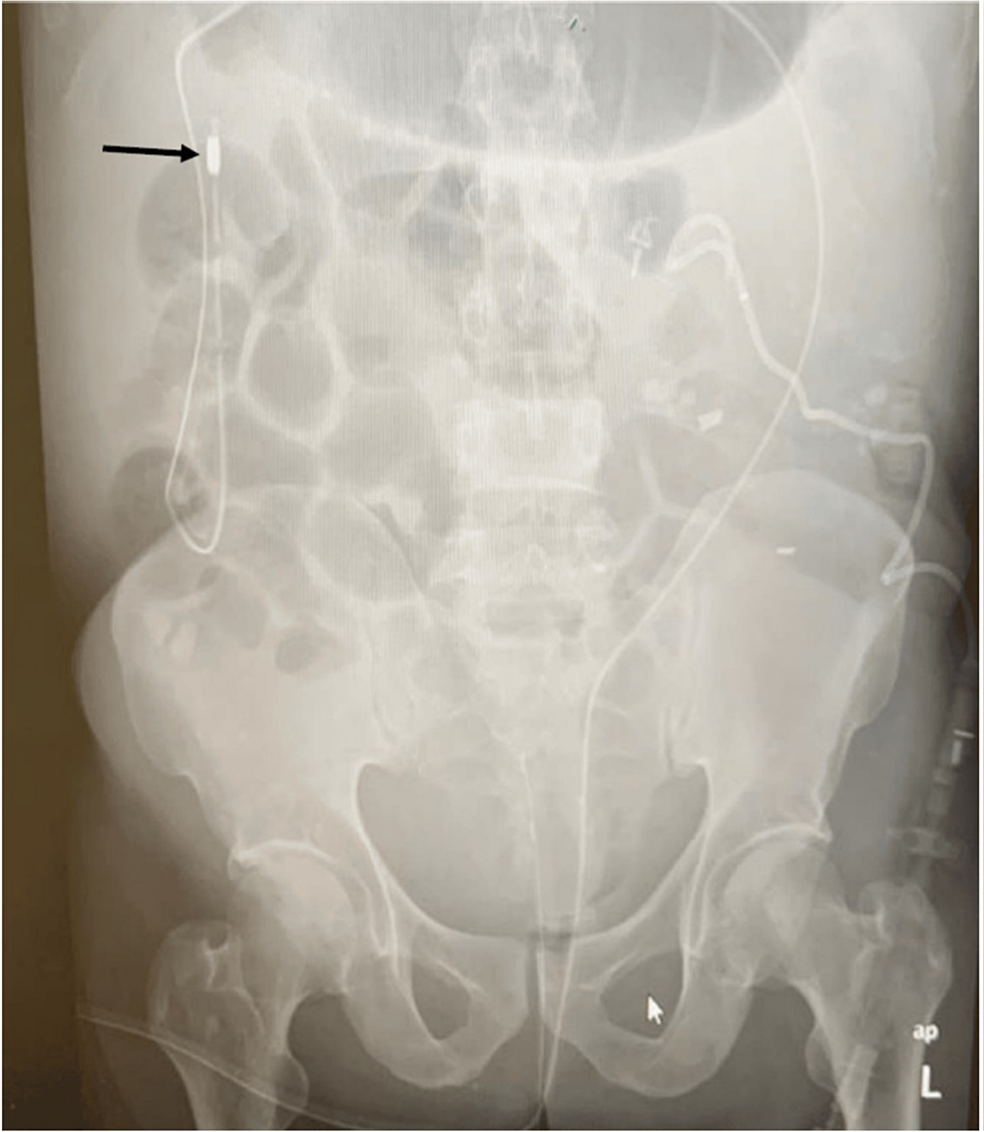
A post-procedure plain abdominal (AP supine view) X-ray. The image shows a significant interval improvement in the gaseous distention of the colon. The Ryle's tube is coiled up from the cecum, and the tip is in the hepatic flexure (line arrow).

Consequently, the patient underwent an emergency colonoscopy for colonic decompression, followed by a gastroscopy without air insufflation, passing through the anus up to 60 cm under fluoroscopy. A dilated colon segment filled with air and gas was encountered and deflated with suctioning. A colonogram displayed a distended lumen, but no definite transition point was identified. With fluoroscopy guidance, a guide wire was placed over the scope. Then, using the guidewire, a customized rectal tube (in this case, a 16F NGT Ryle's tube with increased side holes) was inserted up to the cecum, aspirating 800-1000 mL of liquid stool. A post-procedure abdominal X-ray demonstrated a significant interval improvement in the gaseous distention of the colon, with the rectal tube coiled up from the cecum and the tip visible in the hepatic flexure (Figure 3).

Two days later, the patient tolerated oral intake without nausea or vomiting and passed soft stool, and his abdomen exam showed a soft, non-tender, and not distended abdomen. The Ryle's tube was removed, and the patient was discharged home.

## Discussion

ACPO, also known as Ogilvie's syndrome, poses a significant threat with a near 50% mortality rate if left untreated, with perforation and bowel ischemia constituting the most severe complications [2-5]. Despite ongoing research, the precise etiology and pathogenesis of ACPO remain not entirely elucidated, potentially involving an autonomic nervous system imbalance [6]. This condition is frequently observed in hospitalized patients with underlying comorbidities such as sepsis, trauma, musculoskeletal diseases, and post-surgical situations. Patients experiencing electrolyte imbalances or using medications, such as narcotics and anticholinergics, are at an elevated risk of developing ACPO [4-6].

Various management strategies exist for ACPO, with conservative measures being the initial preference due to their high efficacy in halting disease progression [6]. This entails implementing fasting, initiating proximal gastrointestinal decompression through nasogastric tube suction, placing a decompressive rectal tube, addressing underlying conditions, discontinuing medications that may impact bowel function, correcting electrolyte imbalances, encouraging patient mobility, and altering patient positioning. Conservative management has demonstrated effectiveness in 70-90% of cases [5,6], typically continued for 72 hours with close monitoring. If improvement is not observed or the cecal diameter exceeds 12 cm, a re-evaluation is initiated, potentially involving neostigmine administration and colonic decompression [3-7]. Neostigmine, administered slowly over five minutes, may cause side effects such as bradycardia and bronchospasm, with contraindications including recent myocardial infarction, acidosis, asthma, bradycardia, peptic ulcer disease, and beta-blocker use. Patients not responding to conservative measures or those experiencing ischemia, perforation, or peritonitis may require surgical intervention [3-7].

In comparing conservative and interventional approaches, studies indicate that most patients show improvement with conservative management, underscoring their preference for quality and safety [2]. However, our patient did not respond to conservative management and required endoscopic decompression twice. The next course of action would typically involve surgery; however, we innovatively chose to utilize Ryle's tube as a rectal tube for the first time. This involved placement in the right colon using a guidewire under fluoroscopy, resulting in favorable patient recovery and obviating the need for surgical intervention. Consequently, applying Ryle's tube through the rectum emerges as a novel technique for treating ACPO in non-responsive cases, mitigating recurrence risks, and averting surgical interventions. The flexibility of the tube may reduce the uncommon occurrence of complications such as perforation, making it a promising alternative in the management of ACPO.

## Conclusions

In summary, ACPO poses a life-threatening risk with a substantial mortality rate, especially when not addressed promptly. Although conservative methods are typically favored for their safety and efficacy, there are instances, as observed in our case, where patients do not respond to these treatments, prompting the exploration of alternative strategies. The innovative application of placing Ryle's tube through the rectum into the right colon under fluoroscopic guidance offers a new avenue for addressing unresponsive cases, steering clear of the need for surgical interventions. This method proves effective in efficiently decompressing the colon and preventing recurrence, with a relatively low risk of complications due to the tube's flexible nature.
